# Polyurethane Flexible Joints as an Advanced Adhesive Layer in Sustainable Prefabricated Small Bridge Structures

**DOI:** 10.3390/ma18245659

**Published:** 2025-12-17

**Authors:** Dorota Jasińska, Paweł Szeptyński, Jan Grzegorz Pochopień, Arkadiusz Kwiecień

**Affiliations:** Faculty of Civil Engineering, Cracow University of Technology, 31-155 Cracow, Poland; dorota.jasinska@pk.edu.pl (D.J.); pawel.szeptynski@pk.edu.pl (P.S.); jan.pochopien@doktorant.pk.edu.pl (J.G.P.)

**Keywords:** polyurethane, bridges, flexible joints, composite structures, analytical modeling, finite element analysis

## Abstract

This study presents an analysis of adhesively bonded reinforced concrete composite beams. Experimental results are compared with two computational approaches—an iterative algorithm based on an analytical solution and finite element analysis (FEA)—for simply supported composite beams subjected to four-point bending. The cross-section of the beam consists of two reinforced concrete beams bonded together with different adhesive layers: either flexible polyurethane or a stiff epoxy resin layer. This article begins by outlining the process used to determine the parameters for the flexible materials. The linear analytical model, based on the hypothesis of planar cross-sections for bent components and approximating the behavior of the adhesive layer by the pure shear state, leads to closed-form formulas for deflections and stresses in individual components of the system. These formulas are employed in an iterative procedure to evaluate the post-cracking behavior of composite beams. Conversely, the FEA model accounts for material non-linearity in both the adhesive and concrete, as well as the possibility of decohesion of the adhesive layer, providing a more detailed and accurate representation of the structure. The allowable loads, deflections, and stresses derived from both methods are evaluated and compared across various stages of structural performance: prior to cracking, and two serviceability limit states. The obtained results are validated through comparison with experimental data. The aim of this study is to evaluate the effectiveness of the analytical method for rapid assessment of the capacity of composite concrete structures in different work phases. The iterative procedure based on the analytical solution is found to provide reasonable approximations in terms of the deflection, stress distribution, and crack depth.

## 1. Introduction

Many structures, particularly those constructed several decades ago, face challenges related to aging materials, inadequate maintenance, and evolving safety standards. These aging buildings often exhibit signs of deterioration, such as cracks, corrosion, and structural weakness, necessitating their urgent renovation or even complete reconstruction. In such cases, innovative solutions are essential to enhance the structural integrity and extend the service life of these buildings. Bridge structures are commonly made with steel and concrete elements, using steel connectors between steel beams and cast-on-site concrete slabs [1]. The connection methods for composite bridge structures, particularly focusing on steel connectors, have been extensively analyzed in numerous publications [2,3,4]. To prevent microcracking in the concrete around the connector areas and to accelerate the on-site construction process, new connection technologies based on adhesive bonding solutions have been proposed for both steel-to-concrete [5] and concrete-to-concrete [6] connections. Typically, the use of stiff, high-strength epoxy adhesives is reported; however, this can lead to stress concentration in the bond, causing damage to the connection. To overcome this drawback, the application of flexible polyurethane adhesives (FPUs) as the interface between concrete elements, without any additional connectors, has been proposed. This kind of flexible connection, which transfers high shear loads as well as high deformations, fills the existing gaps in composite bridge connections. These materials are also useful for repairing damaged concrete [7] and connecting old concrete with newly poured layers of concrete [8]. Using FPUs for flexible joints in structural elements, instead of traditional mechanical connectors, can shorten assembly time, enhance work safety, lower operational costs, and reduce environmental impacts. Prefabrication and flexible joints enable quicker and safer assembly, reducing the need for heavy equipment and minimizing road, rail, and pedestrian disruptions.

Polyurethanes have been widely utilized in numerous industrial and commercial fields since the 1940s, with significant applications in the chemical, automotive, textile, and civil engineering industries [9,10,11].

In the study performed in [12], numerous methods for producing urethanes have been discussed. The first attempts to bond concrete-to-concrete components in composite structures were documented in [13,14], and the benefits and drawbacks of using stiff epoxy adhesives for concrete bonding were discussed in [6]. However, these articles did not mention any flexible solutions.

In an article by Kwiecień et al. [15], it was shown that no significant weight loss in FPU is observed for elevated temperatures up to 200 °C. Similar findings have been reported for cross-linked polyurethane elastomers by Król and Pilch-Pitera [16], and for hydroxy terminated polybutadiene polyurethane by Sarkar and Adhikari [17]. Chen et al. [18] have demonstrated that the thermal stability of polyurethane nanocomposites can be improved through the use of sepiolite modified with γ-aminopropyltriethoxysilane. They also found that the loss of tensile strength due to thermal aging was lower in these nanocomposites when compared to pure polyurethane. Pyrolysis of synthetic polyurethane based on isophorone diisocyanate was investigated by Zhang et al. [19]. Raftery et al. [20] have examined the durability of polyurethane-based adhesive joints for wood adherends. Their findings indicated that standard delamination requirements were met, and the shear response of the analyzed joints was comparable to that of control solid wood specimens and resorcinol formaldehyde-bonded wood specimens. Predominantly, failure was observed in the wood itself. De Santis et al. [21] investigated the durability of steel-reinforced polyurethane bonds, concluding that the artificial ageing process did not affect the joint’s bond performance in a significant way. Rutkowska et al. [22] have analyzed the degradation of three types of polyurethanes in sea water. The effect was strongly dependent on the structure of the polymer: while the investigated poly(ester-urethane)A was torn into pieces in a tensile test after 6 months in a natural seawater environment, the tensile strength of the other poly(ether-urea-urethane)s remained almost unaffected after 12 months. It was also shown that degradation of polyurethanes in sea water occurs predominantly due to enzymatic processes, as exposure to sea water with sodium azide (eliminating micro-organisms) did not influence the tensile response of all analyzed polyurethanes. Junco et al. [23] have examined lightweight mortars with the addition of recycled polyurethane foams. While a small reduction in strength was observed after ageing, the recycled mortars could be still considered unaffected by this process when compared to reference specimens. A review on the degradation and stabilization of polyurethane elastomers can be found in [24].

**Figure 1:**
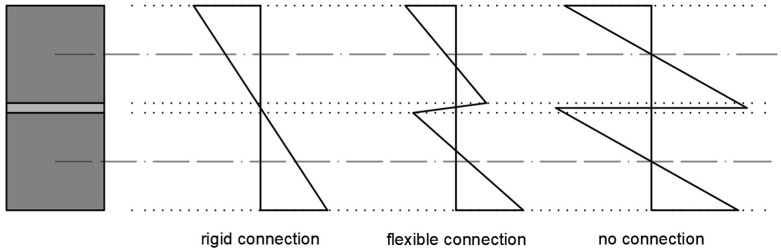
Strain distribution in composite cross-sections, depending on the type of interface connection used [21].

The mechanical behavior of a composite girder structure is significantly influenced by the type of connection used [25], as illustrated in Figure 1. When a rigid connection is used, Bernoulli’s hypothesis regarding plane cross-sections applies to the entire composite section. If no tangential interaction between the top and bottom beams is assumed, each component bends independently. A flexible interface connection, created using a sufficiently flexible adhesive layer (in terms of material properties and geometry), provides a middle ground between a rigid connection and free-sliding. The use of flexible polyurethane (FPU) adhesives generally leads to reduced stress concentrations and ensures their even distribution.

The performance of adhesively bonded composite beams is a subject of ongoing research. A composite girder in the form of a concrete slab with adhesively bonded steel I-section has been analyzed by Bouazaoui et al. [26]. Similar research for various girder variants has been conducted by Jurkiewiez et al. [27]. Nordin and Täljsten tested a concrete beam with a Glass Fiber-Reinforced Polymer (GFRP) I-section beneath it, with an additional CFRP film attached to the bottom flange of the I-section [28]. Timber–Concrete Composites (TCCs) have been investigated by a number of researchers. Kanócz and Bajzecerová [29] examined concrete slabs attached to the bottom of cross-laminated timber (CLT) and variable laminated timber beams. Giv et al. tested plain and reinforced concrete slabs connected with glue-laminated timber using epoxy and polyurethane adhesives [30,31]. Shehada also examined TCC beams manufactured via either dry or wet processes [32]. Various layouts of the adhesive layers in TCCs have been compared by Frohnmüller et al. [33]. Louter analyzed adhesively bonded steel reinforcement of glass beams [34]. Viscoelastic analysis of adhesively bonded CLT beams was investigated in [35]. However, it seems that there is a gap in the literature concerning the adhesive bonding of two reinforced concrete elements bent together.

A flexible connection between a reinforced concrete beam and a reinforced concrete slab was proposed in [36]. In this case, a linear one-dimensional analytical model of a beam was under consideration. Analytical modeling of adhesively bonded beams has been the subject of a number of research papers. The problem of three-point bending of a composite girder consisting of two similar adherends connected by a layer of adhesive was analyzed as a potential testing procedure for determination of the adhesive’s shear modulus by Moussiaux et al. [37]. The authors provided closed-form analytical expressions for deflection as well as for shear stresses in the adhesive layer; however, formulae for normal stresses in the adherends were not given. In fact, the longitudinal deformation of adherends—influencing the magnitude of normal stresses—was not investigated. Similar analyses have been performed by de Morais [38,39], focusing on the problems in which the adhesive was regarded as an ideal elastic–plastic material. Both of these models were limited to three-point bending problems.

This study investigates the behavior of a composite structure consisting of two reinforced concrete beams joined with multiple adhesives across different work phases, employing both analytical and finite element models, with results compared against experimental observations. The novelty of the proposed analytical approach lies in the use of theoretical explicit formulas to determine the values of deformations and stresses in the analyzed structures. This method allows for rapid estimation of the results, which provides a significant advantage compared to the more time-consuming FEM calculations. These theoretical formulas, based on simplified assumptions and approximations, enable preliminary assessment of the structural state (particularly in the design phase), without the need for complex and time-intensive numerical analyses. This approach allows for the rapid generation of approximate results, providing a useful starting point for more detailed calculations and significantly increasing the efficiency of the design process.

The presented research is the preliminary step in a new branch of a broader research activity focused on the design and performance assessment of flexible polyurethane joints. Connecting reinforced-concrete slabs and beams (in particular, prestressed prefabricated beams) by means of adhesive bonding through the use of FPUs is a new research topic.

## 2. Materials and Methods

### 2.1. Adhesive Layers

For the connection between two beams, elastic polyurethane adhesives (PM, PS, PST) and stiff epoxy resin (Sikadur 30) were used. The polyurethane adhesives, distributed by FlexAndRobust Systems (Cracow, Poland), belong to the type P category. These adhesives have been successfully utilized to repair concrete surfaces and enhance the strength of concrete beams and masonry structures, due to their hyperelastic properties. Detailed mechanical properties of these FPUs, such as their Young’s moduli, were obtained through tests, the results of which have been described in [40,41,42]. Additional information on the material properties of these polyurethanes can be found in [43,44]. The stiff layer, made of Sikadur 30 resin (produced by SIKA) was used for comparison. In this paper, the Sikadur 30 epoxy resin will be denoted as “E.” The properties of the epoxy resin are presented, in accordance with the producer’s data, in Table 1.

### 2.2. Experimental Configuration and Testing Methods

**Figure 2:**
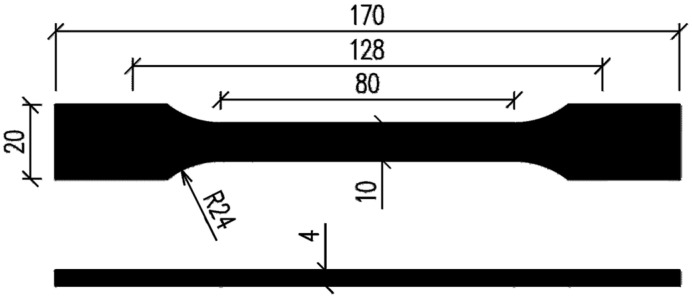
Geometry of type 1A specimen as per ISO 527 standard.

The analyzed flexible polyurethane adhesives tend to change their properties, depending on the strain rate of the applied load [36]. This means that it is not possible to determine e.g., a single value for the Young’s modulus of these materials as input to analytical formulas and the Abaqus/CAE 2022 software in the case of purely elastic analysis. Flexible Polyurethane (FPU) materials exhibit mechanically non-linear and viscoelastic behavior. In order to examine the properties of these materials, tests were carried out in accordance with ISO 527 [45,46], performing a uniaxial tension test using a Zwick/Roell 1455 20 kN testing machine (Zwick Roell Polska, Wrocław, Poland) at the Cracow University of Technology laboratory, utilizing shape 1A specimens (Figure 2).

**Figure 3:**
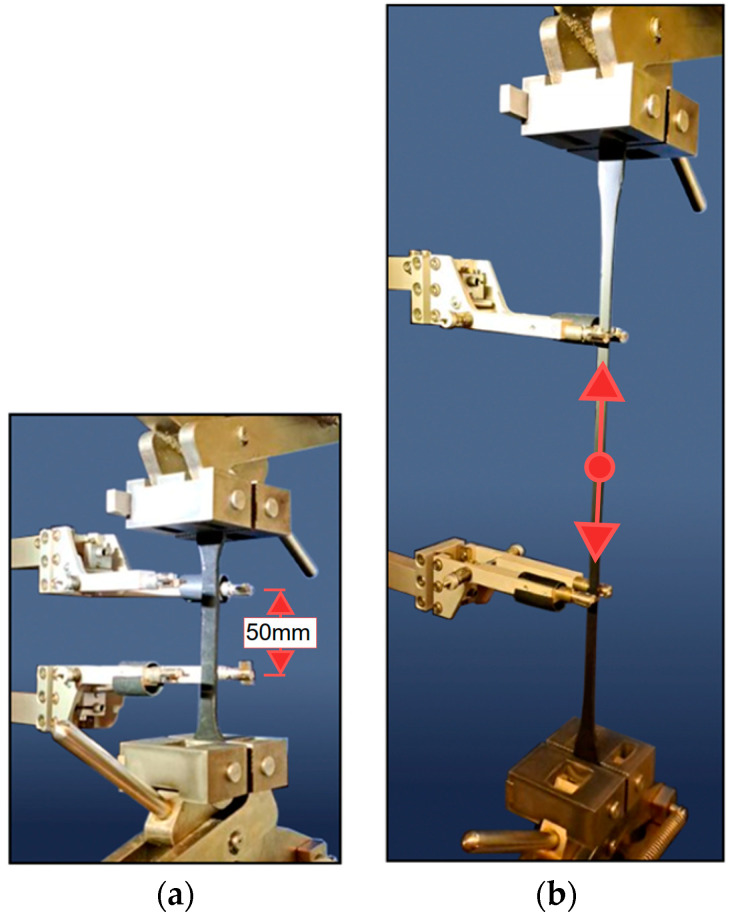
Tensile test arrangement: (**a**) prior to specimen deformation; (**b**) after specimen deformation.

Tests have been performed under constant temperature conditions (23 °C) for 7 different FPUs [47], including those used in this paper (F&R: PS, PTS, and PM). The tests were programmed for variable rates of strain (10^−3^, 10^−2^, 10^−1^, 1, and 10 [1/min]), using a long-distance extensometer with a 50 mm base (see: Figure 3). Six specimens for each material and each ratio were used. The tensile test is presented in Figure 3, with a specimen before and after deformation (just before rupture).

### 2.3. Concrete and Reinforcement

The concrete was assumed to be of class C30/37. The material properties, in accordance with the European design standard [48], are as follows: mean cylinder compressive strength $f_{cm}=38\;MPa$, mean tensile strength of the concrete ${\;f}_{ctm}=2.9\;MPa$, mean Young’s modulus $E_{cm}=32.0\;GPa$, Poisson’s ratio $\nu_{c}=0.2$, compressive strain at the peak stress ${\;f}_{c}$ ${\;\varepsilon }_{c1}=2.2\;‰$, and ultimate compressive strain ${\;\varepsilon }_{cu1}=3.5\;‰$.

Reinforcement bars made of class AIIIN and grade B500SP steel were adopted. The mechanical properties of the steel were also taken based on the European design standard [48], as follows: mean Young’s modulus $E_{s}=200\;GPa$ and Poisson’s ratio $\nu_{s}=0.3$. The reinforcement’s limit yield stress was $f_{y}=500\;MPa$.

### 2.4. Case Study

**Figure 4:**
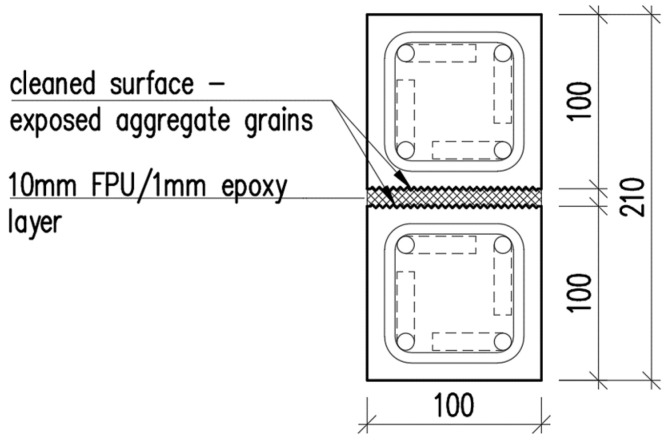
Cross-section of the composite girder.

**Figure 5:**
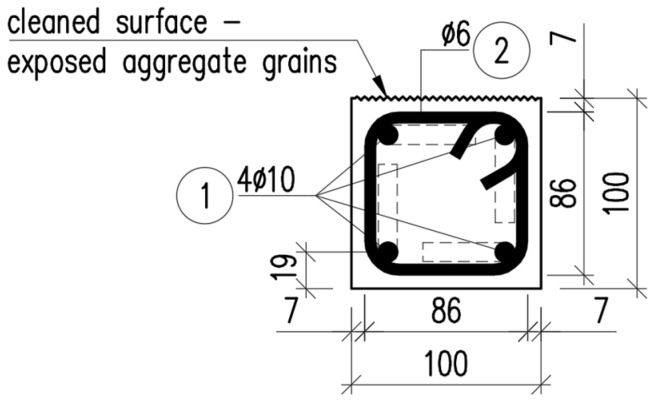
Cross-section of the RC beam.

**Figure 6:**
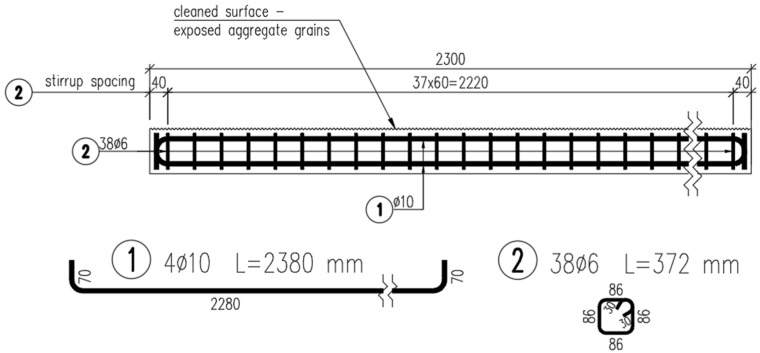
Longitudinal section of the beam and its reinforcement.

A case study considering a composite girder was conducted in order to assess how the mechanical properties of the flexible FPUs influence the mechanical behavior of composite structures. The composite girder comprised two reinforced concrete (RC) beams with a square cross-section measuring 100 mm × 100 mm, joined together with the use of either 10 mm thick FPU layer or an epoxy layer of thickness 1 mm (see: Figure 4). The span length of the symmetric, simply supported composite girder was L=2 m. The concrete elements were reinforced with steel rebars, as presented in Figure 5 and Figure 6. The concrete cover was 10 mm thick, measured from the edge of the concrete to the outer edge of the stirrup. The main reinforcement consisted of four bars of ϕ=10 mm diameter. The stirrups were two-legged with a diameter of $\phi_{w}=6\;mm$.

**Figure 7:**
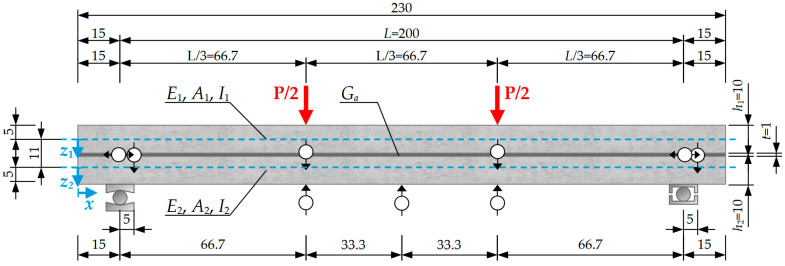
Diagram of the analyzed composite beam—dimensions in cm.

The four-point bending performance of the girder was examined (Figure 7). Three deformation phases were distinguished in the comparative analysis of theoretical prediction, numerical simulation and experimental results. The first range of deflection (Phase I) is the limit of an uncracked reinforced concrete cross-section. The next two stages are defined by deflections specifying the serviceability limit states equal to L/500 (Phase II) and L/250 (Phase III), respectively.

### 2.5. Preparation of the Experimental Setup

**Figure 8:**
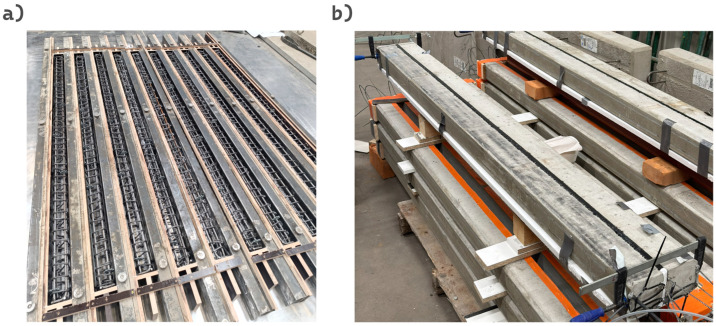
(**a**) Reinforcement of the beams prepared in the formwork; (**b**) Pair of beams after casting the joint.

The design of the prefabricated beams was developed with consideration of structural strength, geometric constraints, and reinforcement layout requirements according to standard EN1992-1-1. Based on the completed design documentation, the beams were manufactured at a precast concrete fabrication facility (Figure 8a). Next the prefabricated elements were subjected to a curing period of at least 30 days, under controlled environmental conditions, ensuring protection from atmospheric exposure and preventing uncontrolled moisture or temperature fluctuations. This procedure was implemented to stabilize the mechanical properties of the concrete and to minimize the influence of shrinkage and time-dependent (rheological) deformations on the subsequent structural testing results.

After the concrete curing process, the beams were coupled in pairs for adhesive bonding. The bonding surfaces were thoroughly cleaned to remove dust, loose particles, and any residual contaminants, ensuring adequate substrate preparation. Beam pair were bonded using four different adhesive materials: an epoxy resin (Sikadur^®^ 30) and three types of polyurethane adhesives (F&R^®^ PS, F&R^®^ PM, and F&R^®^ PST). Prior to adhesive bonding with the use of FPUs, a uniform layer of F&R ZP Primer was applied to promote proper adhesion and to enhance the mechanical interlock between the adhesive and the concrete substrate. The thickness of the adhesive layer for the epoxy resin (approximately 1 mm) was controlled by pressing the beam elements together, whereas for the polyurethane adhesives, 10 mm spacers were used to maintain a uniform joint thickness (Figure 8b).

**Figure 9:**
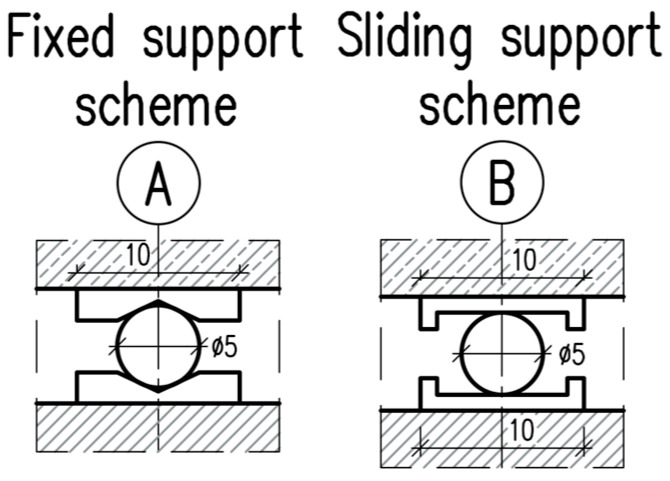
Supports—dimensions in cm. A axis—pinned support. B axis—roller support.

Th components of the test rig were assembled, including the load-transfer hinges, supports (see: Figure 9), aluminum plates, and sensor mounts. The mounts were bonded to the beam surfaces using two component epoxy Poxipol adhesive. The supports were made of steel plates with a width of 100 mm. Under the concentrated load application points, 80 mm steel wide plates were installed to mitigate stress concentrations and prevent local crushing of the concrete.

The beam assemblies were subsequently positioned on the testing frame in strict accordance with the predefined experimental configuration. Inductive displacement transducers (HBM WA series) were installed at the designated measurement locations as specified in the instrumentation layout (see: Figure 8). Prior to testing, all inductive sensors underwent functional verification to ensure proper calibration and signal stability. The measurement system enabled continuous monitoring of midspan and support-region deflections under concentrated loading.

The final stage of preparation involved configuring the Instron PL250N testing machine, including the parameters of loading rate, maximum load range, and data acquisition settings. The machine provides a maximum load capacity of 250 kN. The sampling frequency for the HBM WA inductive displacement transducers was set to 2 Hz.

### 2.6. Simplified Analytical Model

The analytical solution of the analyzed girder was based on the model of a multi-layer composite beam proposed in [49], assuming separate longitudinal displacement for each bent composite layer but one common deflection for the composite. This linear theory presupposes that the girder is constructed from alternating layers of bent beams and adhesive layers. The bent beams are modeled according to the Bernoulli–Euler theory while the adhesive layers, due to their significantly lower rigidity, are assumed to work in a state of pure shear. Closed-form design formulas for deflections and stresses may be derived from analytical solutions obtained with the use of the considered model under various boundary and loading scenarios [50].

#### 2.6.1. System of Governing Equations

For a girder consisting of two bent beams and a single adhesive sheared layer, the resulting governing Equation (1) are as follows:

(1) $$\left\{\begin{array}{l} \frac{d^{2}{\tilde{u}}_{1}}{dx^{2}}+\beta \left[\left({\tilde{u}}_{2}-{\tilde{u}}_{1}\right)+\alpha \frac{d\tilde{w}}{dx}\right]=0\\ \frac{d^{2}{\tilde{u}}_{2}}{dx^{2}}-\gamma \left[\left({\tilde{u}}_{2}-{\tilde{u}}_{1}\right)+\alpha \frac{d\tilde{w}}{dx}\right]=0\\ \frac{d^{4}\tilde{w}}{dx^{4}}=\eta +\delta \left[\left(\frac{d{\tilde{u}}_{2}}{dx}-\frac{{d\tilde{u}}_{1}}{dx}\right)+\alpha \frac{d^{2}\tilde{w}}{dx^{2}}\right] \end{array}\right.,$$

where the unknown functions ${\tilde{u}}_{1},{\tilde{u}}_{2},\;\tilde{w}$ represent the non-dimensional displacements: ${\tilde{u}}_{1}=\frac{u_{1}}{L}$ and ${\tilde{u}}_{2}=\frac{u_{2}}{L}$ are the longitudinal displacements of the top and bottom beam, respectively, and $\tilde{w}=\frac{w}{L}$ denotes the common deflection, where L is the system’s characteristic length (in the discussed case, a span-length).

The coefficients in Equation (1) are the system’s similarity numbers (2):

(2) $$\alpha =\frac{h_{1}+h_{2}}{2L},\;\;\beta =\frac{G_{a}L^{2}b}{E_{1}A_{1}t},\;\;\gamma =\frac{G_{a}L^{2}b}{E_{2}A_{2}t},\;\;\delta =\frac{G_{a}L^{3}b\left(\frac{h_{1}}{2}+\frac{h_{2}}{2}+t\right)}{t\left(E_{1}I_{1}+E_{2}I_{2}\right)},\;\eta =\frac{qL^{3}b}{\left(E_{1}I_{1}+E_{2}I_{2}\right)},\;\;\lambda =\sqrt{\alpha \delta +\beta +\gamma },$$

where b is the girder width; t is the adhesive layer’s thickness; q is the line load resulting from the self-weight of the girder and possible external uniformly distributed load; the quantities with subscripts i=1 and i=2 refer to the top and bottom beams, respectively; $h_{i},\;A_{i},\;I_{i},\;E_{i}$ denote the heights, areas, second moments of the area, and the Young’s moduli of the top beam (i=1) and the bottom beam (i=2), respectively; and $G_{a}$ represents the Kirchhoff’s modulus of the adhesive. The inhomogeneous linear fourth-order system of the ordinary differential Equation (1) can be converted into a set of eight first-order equations. Such a system can be solved analytically and closed-form formulas can be determined for deflections, longitudinal strains, and stresses under various support conditions and load combinations of the girder, where the forms of these formulas depend on the boundary conditions and the loading.

#### 2.6.2. Analytical Solutions for the Four Point Bending of a Simply-Supported Beam Considering Self-Weight

In the case of a simply supported girder subjected to the combination of a uniformly distributed load (self-weight) and a system of two symmetric point loads P2 (see Figure 7), the maximum deflection $w_{max}$ and the longitudinal strain distributions $\varepsilon_{i}\left(z_{i}\right)$ along the beams’ heights in the midpoint of the girder span are superpositions of the solutions for both loading cases [50], as presented in Equation (3).

(3) $$\begin{array}{c} w_{max}=w_{max.q}+w_{max,P}\\ \varepsilon_{i}(z_{i})=\varepsilon_{i,q}(z_{i})+\varepsilon_{i,P\;\;}(z_{i})\;\;\;\;for\;i=1,2 \end{array}\;,$$

in which the components from the self-weight are defined by Equations (4)–(6) as follows:

(4) $$w_{max,q}=\eta L\frac{768\alpha \delta e^{\frac{\lambda }{2}}+\left(e^{\lambda }+1\right)\left[5\lambda^{4}\left(\beta +\gamma \right)+48\alpha \delta (\lambda^{2}-8)\right]}{384\;\lambda^{6}(e^{\lambda }+1)},$$

(5) $$\varepsilon_{1,q}\left(z_{1}\right)=\eta \left[-\alpha \beta \left(\frac{2e^{\frac{\lambda }{2}}}{\lambda^{4}\left(e^{\lambda }+1\right)}+\frac{\lambda^{2}-8}{8\lambda^{4}}\right)-\frac{z_{1}}{L}\left(\frac{\alpha \delta \left(2e^{\frac{\lambda }{2}}-e^{\lambda }-1\right)}{\lambda^{4}\left(e^{\lambda }+1\right)}-\frac{\beta +\gamma }{8\lambda^{2}}\right)\right],$$

(6) $$\varepsilon_{2,q}\left(z_{2}\right)=\eta \left[\alpha \gamma \left(\frac{2e^{\frac{\lambda }{2}}}{\lambda^{4}\left(e^{\lambda }+1\right)}+\frac{\lambda^{2}-8}{8\lambda^{4}}\right)-\frac{z_{2}}{L}\left(\frac{\alpha \delta \left(2e^{\frac{\lambda }{2}}-e^{\lambda }-1\right)}{\lambda^{4}\left(e^{\lambda }+1\right)}-\frac{\beta +\gamma }{8\lambda^{2}}\right)\right],$$

whereas the components from the four-point bending part take the form of Equations (7)–(9):

(7) $$w_{max,P}=PL^{3}\frac{\lambda^{3}\left(\beta +\gamma \right)\left(e^{\lambda }+1\right)\left(3\xi -4\xi^{3}\right)+24\alpha \delta \left[e^{\lambda \left(\frac{1}{2}-\xi \right)}-e^{\lambda \left(\frac{1}{2}+\xi \right)}+\lambda \xi \left(e^{\lambda }+1\right)\right]}{48\lambda^{5}\left(E_{1}I_{1}+E_{2}I_{2}\right)\left(e^{\lambda }+1\right)},$$

(8) $$\varepsilon_{1,P}=\frac{PL}{2}\frac{\alpha \left(\delta z_{1}+\beta L\right)\left(e^{\lambda \left(\frac{1}{2}+\xi \right)}-e^{\lambda \left(\frac{1}{2}-\xi \right)}\right)+\lambda \xi \left(e^{\lambda }+1\right)\left[\left(\beta +\gamma \right)z_{1}-\alpha \beta L\right]}{\left(E_{1}I_{1}+E_{2}I_{2}\right)\lambda^{3}\left(e^{\lambda }+1\right)},$$

(9) $$\varepsilon_{2,P}=-\frac{PL}{2}\frac{\alpha \left(\delta z_{2}+\gamma L\right)\left(e^{\lambda \left(\frac{1}{2}+\xi \right)}-e^{\lambda \left(\frac{1}{2}-\xi \right)}\right)-\lambda \xi \left(e^{\lambda }+1\right)\left[\left(\beta +\gamma \right)z_{2}-\alpha \gamma L\right]}{\left(E_{1}I_{1}+E_{2}I_{2}\right)\lambda^{3}\left(e^{\lambda }+1\right)}.$$

In Formulas (4)–(9), $z_{1,2}$ are the vertical distances of the chosen points in the cross-section from the neutral bending axis (Figure 7), while ξ = *x*/*L* is the non-dimensional distance of the point of application of the point-load from the support. Longitudinal stresses can be easily determined from strains. Their extreme values in the *i*-th bent beam are obtained using Equation (10):

(10) $$\sigma_{i}^{max/min}=E_{i}\varepsilon_{i}\left(z_{i}=\pm \frac{h_{i}}{2}\right).$$

#### 2.6.3. Application of the Analytical Solution to Reinforced Concrete

The modeling of reinforced concrete bent elements requires considering the non-linear behavior of the concrete material, particularly taking into account progressive cracking, which reduces the beam’s flexural stiffness. The analytical model cannot incorporate the localization of cracks in the concrete. However, for the analytical calculation of deflections in cracked beams, it is common practice to adopt a constant reduction in flexural stiffness for the entire beam [51]. Thus, Formulas (3)–(9), derived from the linear analytical model, can also be employed to iteratively solve the non-linear deformation problem of a cracked beam.

For reinforced concrete beams, the weighted cross-sectional properties ($A_{i},I_{i})$ are determined by adjusting the contributions of materials according to the ratio of their Young’s moduli.

**Figure 10:**
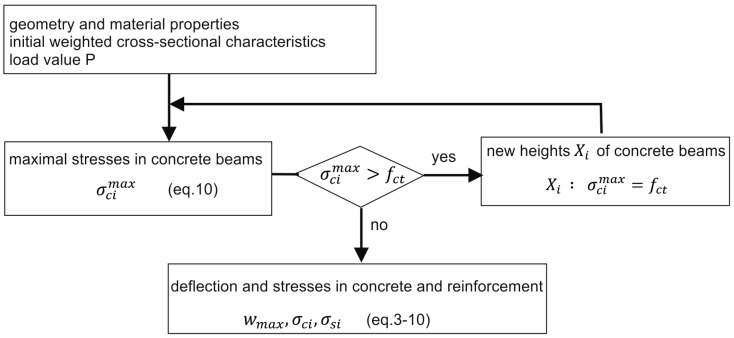
Iterative process diagram.

At the start of the iteration loop, the cross-sectional characteristics of the beams are determined for the entire heights of the concrete beams ($h_{1},h_{2})$. If the tensile stress in the concrete exceeds the mean tensile strength, new characteristics are determined, considering only the part of the concrete section where the strength has not been exceeded. The heights of the active zones of both concrete cross-sections ($X_{1},X_{2})$ are determined in an iterative manner, as presented in Figure 10. The condition for terminating the iteration process is that the change in the cross-sectional characteristics determined in subsequent iterations is below the accepted accuracy. The updated cross-sectional characteristics allow for the determination of beam deflection, as well as axial strains and stresses at the mid-span of the beam, in accordance with Equations (3)–(10).

### 2.7. Finite Element Method Model for a Simply Supported Beam Under Point Loads

To validate the accuracy of the linear (iteratively) analytical model, finite element analyses comparing the loading processes of composite girders bonded with different FPUs were carried out using the Abaqus software [52].

#### 2.7.1. Geometry, Supports, and Loads

**Figure 11:**
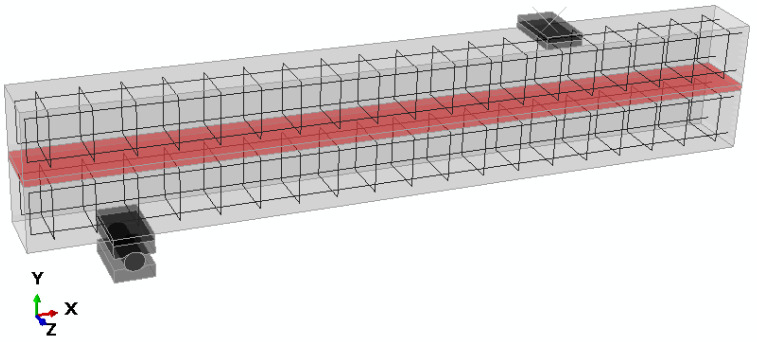
Finite element model of the half of the girder.

The three-dimensional finite-element model precisely reflected the geometry, support placement, and reinforcement layout of the original design of the composite girder, as shown in Figure 11. Several sources of non-linearity were accounted for: non-linear material behavior (of FPU, concrete, and reinforcement steel), and contact at the support zone. Due to the symmetry of the problem, only half of the composite girder was modeled.

The reinforcement was represented explicitly as individual bar elements, implemented as stringers tied to the edges of the three-dimensional concrete solid elements. The adhesive interface was modeled using three-dimensional brick elements. The roller support was idealized as an assembly of rigid bodies in contact, permitting both translation and rotation. The concentrated loads in the four-point bending configuration were applied by prescribing vertical displacements at the loading points.

#### 2.7.2. Mechanical Characteristics of Materials

FPUs are considered hyperelastic and were modeled using the Mooney–Rivlin form of strain energy potential, with coefficients derived from the uniaxial tension test data and compressibility defined by the Poisson’s ratio ν=0.4, as referenced in [53,54]. The comparative epoxy layer was assumed to be linear elastic with Young’s modulus assumed according to Table 1 and Poisson’s ratio equal to 0.48. The concrete damage plasticity (CDP) constitutive model was selected to characterize the inelastic behaviors of concrete, encompassing tensile cracking and compressive crushing, which incorporates the principles of isotropic damage elasticity and isotropic tensile and compressive plasticity. In addition to constitutive parameters defining the yield surface and plastic flow (Table 2), it was also necessary to provide tabular data defining post-critical compressive and tensile behaviors and scalar damage parameters, thus defining irreversible damage occurring during the fracturing process. In the present work, the non-linear compressive behavior of concrete was assumed, according to Eurocode 2 [48], to follow Equation (11)—compressive stress $\sigma_{c}$ is calculated as follows:

(11) $$\begin{array}{ccc} \sigma_{c}=f_{cm}\frac{k\eta -\eta^{2}}{1+(k-2)\eta } & for & \sigma_{c}\geq 0.4f_{cm}, \end{array}$$

where $\eta =\frac{\varepsilon_{c}}{\varepsilon_{c1}}$ and $k=1.05E_{cm}\frac{\varepsilon_{c1}}{f_{cm}}$.

Meanwhile, the post-cracking tensile constitutive model was adopted, after [55], using Equation (12)—tensile stress $\sigma_{t}$ is equal to:

(12) $$\sigma_{t}=f_{ctm}\frac{x}{\alpha_{t}{\left(x-1\right)}^{1.7}+x}\;\mathrm{for}\;x\geq 1,$$

where $x=\frac{\varepsilon_{t}}{\varepsilon_{cr}}$, $\varepsilon_{cr}=\frac{f_{ctm}}{E_{cm}}$, and $\alpha_{t}=0.312f_{ctm}^{2}$.

The resulting tabular stress-crushing/cracking (inelastic) strain data for C30/37 concrete are collated in Table 3. In the above equations $E_{cm}$ stands for mean Young’s modulus of concrete, $f_{cm}$ and $f_{ctm}$ denote mean compressive strength and mean tensile strength of concrete, respectively, and $\varepsilon_{c}$ is compressive strain in concrete, $\varepsilon_{c1}$ is strain at maximum stress, $\varepsilon_{t}$ is tensile strain in concrete.

The steel reinforcement material was assumed to be elastic–perfectly plastic.

## 3. Results

### 3.1. Outcomes of the Polyurethanes Uniaxial Tension Test

**Figure 12:**
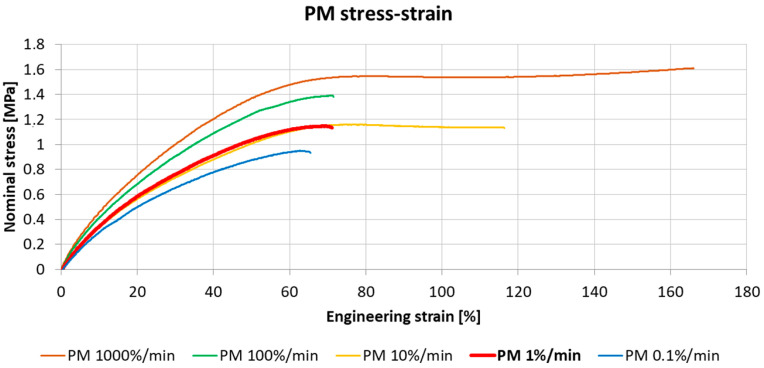
The stress–strain curves for polyurethane PM under different strain rates (mean values).

Based on uniaxial tensile tests, the material properties of the analyzed polyurethanes (PM, PS, and PTS) were assessed across five different strain rate ranges. The tested FPU viscoelastic materials presented increased stiffness at higher strain rates. Figure 12 presents the average stress–strain curves for the polyurethane PM at various strain rates, obtained in the uniaxial test according to the relevant ISO standard [45,46].

**Figure 13:**
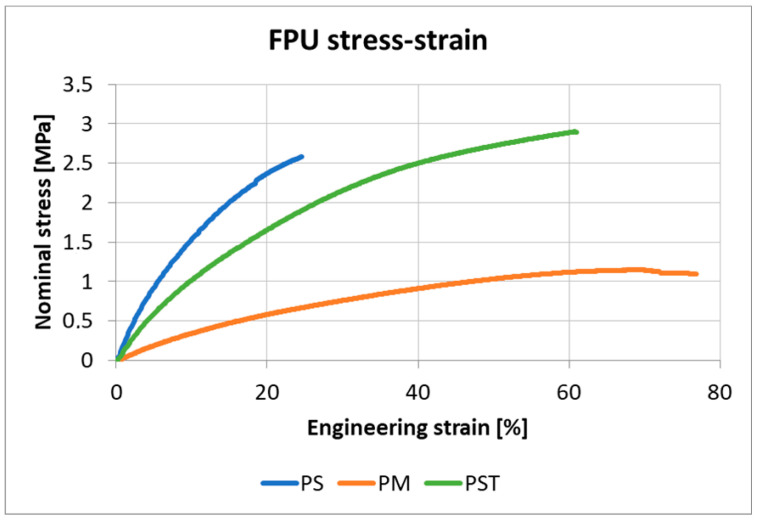
The stress–strain curves for analyzed polyurethanes at a strain rate of 1%/min (mean values).

The curve obtained at a strain rate of 1%/min was used for the calculations. Figure 13 illustrates the average stress–strain curves for the three tested polymers at a strain rate of 1% per minute (10^−2^/min).

The initial tangent Young’s moduli of the polyurethanes were determined from the obtained mean stress–strain curves. These moduli were calculated by finding the slope of a line tangent to each curve at zero strain, disregarding the initial data points influenced by the setup deformation. The Young’s moduli for these initial FPUs (Table 4) were adopted for the analytical model calculations. The parameters defining hyperelastic FPU behaviors in the FEM comparative analysis were calibrated based on the uniaxial tension test data obtained at the strain rate of 1%/min (Figure 12).

### 3.2. Results of the Analytical Model

The composite beams incorporating FPU and epoxy resin adhesive layers were analyzed using the iterative procedure outlined in Section 2.6.3. The three phases of the analysis can be defined using closed-form formula (3) for girder deflection and longitudinal strains in bent beams, as follows:

- Phase I—initiation of cracking in concrete:
  find P for which $max\left[{E_{cm}\varepsilon }_{1}\left(z_{1}=0.5h_{1}\right),\;\;{E_{cm}\varepsilon }_{2}(z_{2}=0.5h_{2})\right]=f_{ctm};$
- Phase II—serviceability limit state L/500
  find (P, $X_{1},\;X_{2})$ for which $w_{max}=L/500;$
- Phase III—serviceability limit state L/250
  find (P, $X_{1},\;X_{2})$ for which $w_{max}=L/250;$

The results of the analyses using the analytical model are summarized in Table 5, including deflections and magnitudes of applied point loads, maximal compressive stresses in concrete, and maximal tensile stresses in reinforcement in the consecutive calculation phases.

### 3.3. Results of the FEM Analysis

After conducting numerical simulations of the incremental loading process of the composite beams using the three-dimensional non-linear FEA model described in Section 2.6, the same quantities were determined (as compiled in Table 6) for the consecutive calculation phases: Deflections, applied loads, compressive stresses in the top fibers at mid span of the upper beam, and tensile stresses at mid span of the bottom reinforcement of the lower beam.

For the purpose of estimating the impact of accounting for the joints’ flexibility on the load-bearing capacity of composite beams, two additional reference solid reinforced-concrete beams representing extreme cases were considered: (a) two beams with 10 × 10 mm cross-sections lying on each other and sliding without friction and (b) two such beams tied together (one beam with double height). These cases define the lower and upper boundaries of the composite beam solutions.

**Figure 14:**
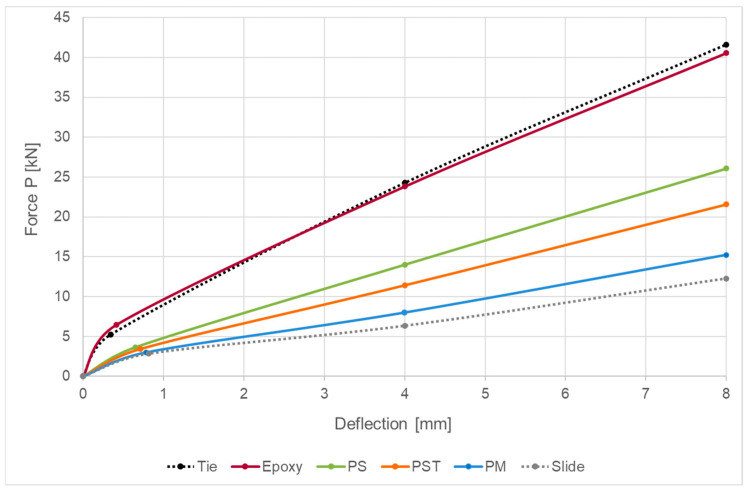
Deflection–load curves obtained with the FEA model for all analyzed girders.

The load–deflection curves obtained with the FEA model for the four analyzed composite beams and two reference beams mentioned above are presented in Figure 14. Points on the curves corresponding to the defined calculation phases are marked with symbols.

### 3.4. Experimental Results

The loading procedure commenced with the zeroing of the testing machine, during which the displacement was set to 0 mm under an applied load of approximately 0 N. This step ensured that all subsequent measurements were referenced to the initial, unloaded state of the beam.

The first loading stage encompassed a displacement range of 0 to 0.7 mm (up to 0.4 mm for epoxy adhesive joints). The loading speed rate was set at 0.1 mm/min to ensure quasi-static application of force and to allow precise monitoring of the initial deformation behavior. The second loading stage encompassed a displacement range of 0.7 to 8 mm, with an increased loading rate of 0.5 mm/min. During this stage, the beam deformations exhibited pronounced nonlinear behavior, and the redistribution of stresses within the beam pair could be observed.

In regard to the deformation phases distinguished in the comparative analysis, Phase I was difficult to be observed unambiguously—relatively large diameters of stirrups as well as relatively large span length (compared to the beams’ self-weight) resulted in very early development of very small cracks.

**Figure 15:**
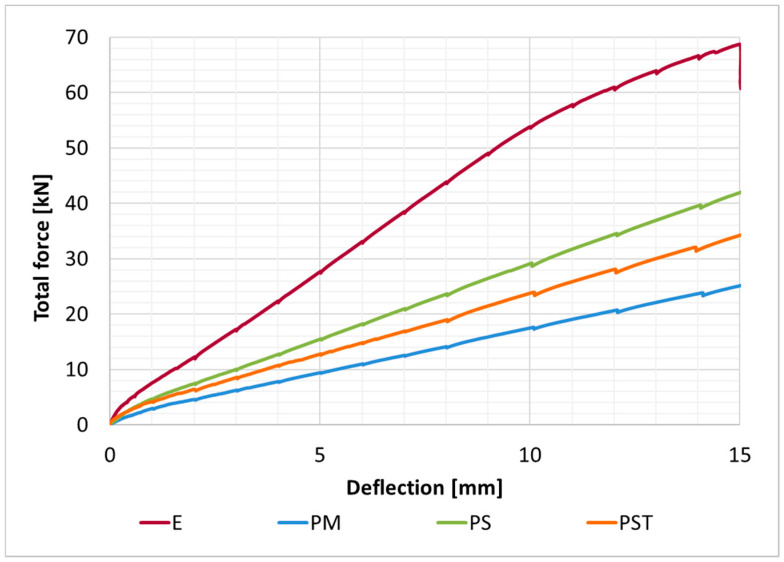
Load–deflection curves recorded during the experiment.

However, tangent stiffness was estimated in each point of the load-displacement curve. For highly deformable polyurethane adhesives it was observed, that within deflection range 0–8 mm generally two characteristic deformation ranges could be distinguished according to the character of changes in tangent stiffness. Initially the stiffness decreases continuously. Then a repeated pattern of sudden drops and subsequent recovery of stiffness may be observed (cf. Figure 15), with the average stiffness remaining approximately constant. These sudden drops are interpreted as individual cracking events [56]. The beginning of this second section in the process of change in tangent stiffness is interpreted here as the end of the Phase I. Such an estimate of the end of the Phase I could not be performed for the beams joined with an epoxy adhesive due to insufficient accuracy of determination of tangent stiffness, what is related with high rigidity of epoxy bondline and resulting small deflections.

The second stage of loading (with displacement rate of 0.5 mm/min) corresponds with Phases II and III.

**Figure 16:**
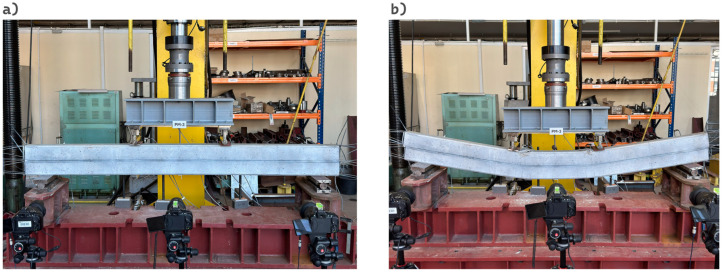
(**a**) initial configuration; (**b**) final configuration (after exceeding phase III).

Load–displacement curves recorded for the four analyzed specimens are presented in Figure 15, and the corresponding deflection and load values at the selected stages are summarized in Table 7. The initial and final configurations of the composite beam with PM adhesive are shown in Figure 16.

### 3.5. Comparisons of the Results

The estimation of the accuracy of the analytical method and numerical calculations with respect to the experiment is presented in Table 8, based on a comparison of load values at the individual phases.

To examine the consistency between the two calculation methods, Table 9 presents a comparison of the compressive stresses in concrete and the tensile stresses in reinforcement determined using the analytical method and the FEM.

**Figure 17:**
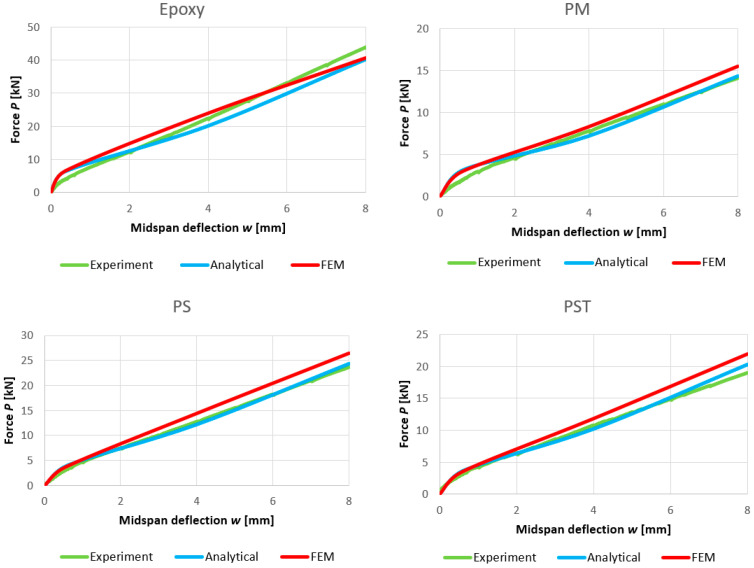
Comparison of load–deflection curves obtained with analytical and numerical models.

Graphs comparing analytical and numerical load–displacement curves for all analyzed composite beams are presented in Figure 17.

**Figure 18:**
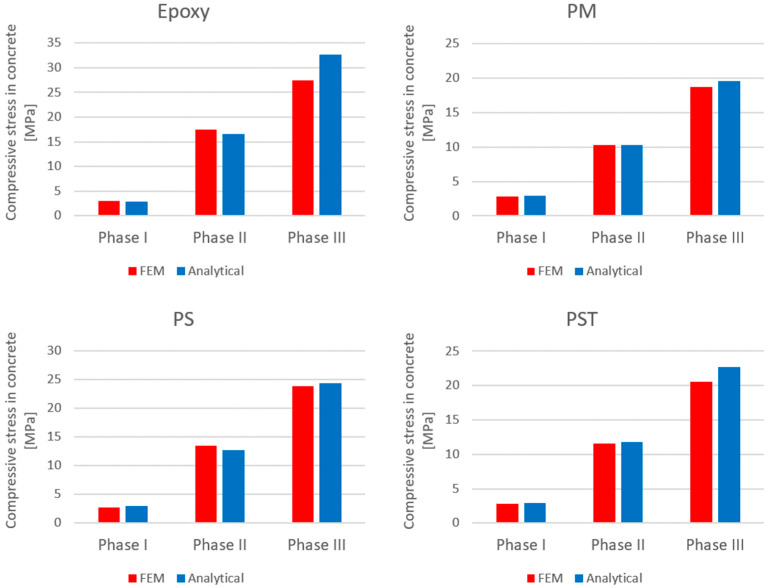
Comparison of compressive stresses in the top fibers at mid-span of the upper beam, obtained with analytical and numerical models.

**Figure 19:**
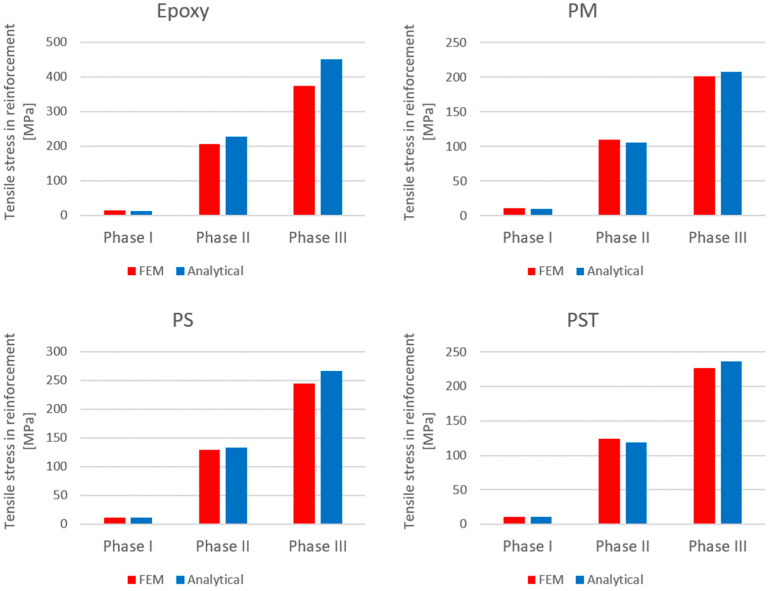
Comparison of maximal tensile stresses in the bottom reinforcement of the lower beam, obtained with analytical and numerical models.

Comparisons of the compressive stresses in the top fibers at the mid-span of the upper beam and tensile stresses in the bottom reinforcement at the mid-span of the lower beam are presented in Figure 18 and Figure 19, respectively.

**Figure 20:**
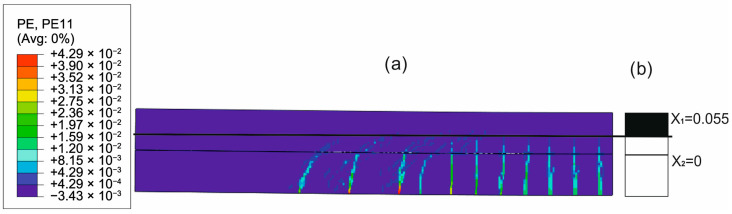
Crack distribution in FEM solution (**a**) versus uncracked beam heights in analytical solution (**b**) in Phase III (deflection L/250) for epoxy adhesive case.

**Figure 21:**
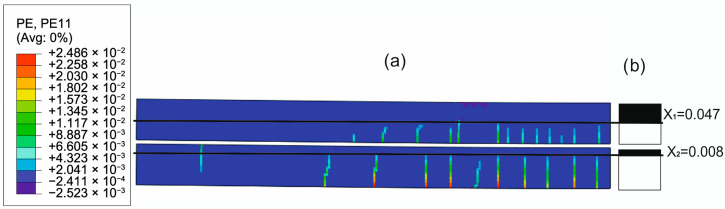
Crack distribution in FEM solution (**a**) versus uncracked beam heights in analytical solution (**b**) in Phase III (deflection L/250) for PS adhesive case.

**Figure 22:**
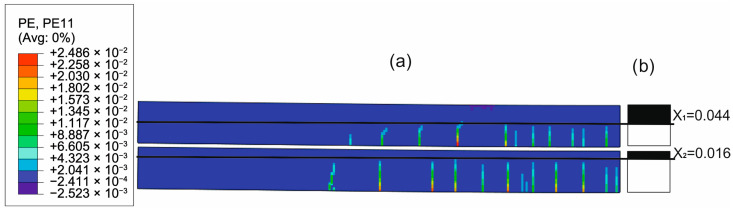
Crack distribution in FEM solution (**a**) versus uncracked beam heights in analytical solution (**b**) in Phase III (deflection L/250) for PST adhesive case.

**Figure 23:**
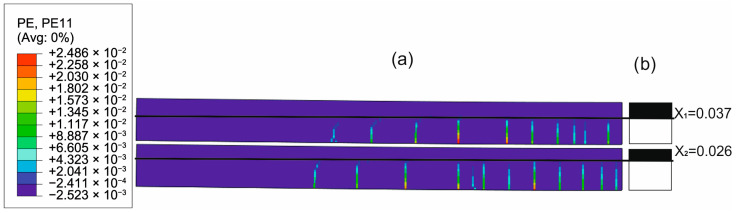
Crack distribution in FEM solution (**a**) versus uncracked beam heights in analytical solution (**b**) in Phase III (deflection L/250) for PM adhesive case.

To evaluate the ability of the analytical model to approximate the concrete cracking process through the determined constant effective height of the working concrete zone, the distributions of cracks in concrete obtained from the FEM solutions were compared with the uncracked heights of both beams obtained with the analytical solutions. Figure 20, Figure 21, Figure 22 and Figure 23 present such comparisons for all analyzed cases in Phase III.

## 4. Discussion

The flexibility of the joint is crucial for accurate estimation of the mechanical response of the considered system (cf. Figure 14 and Figure 15). Using stiffer adhesives or a thinner adhesive layer leads to reduced deflection; this dependency was confirmed by both the analytical model and finite element analysis [47].

The comparison of the analytical, numerical and experimental results (presented in Table 8 and Table 9 and Figure 17, Figure 18, Figure 19, Figure 20, Figure 21, Figure 22 and Figure 23) indicates the following:

- Both analytical and numerical estimates are in good agreement with experimental records. Both these approaches seem to underestimate the deflection at Phase I. It may be stated that analytical model usually predicts higher load at first cracking, while numerical simulation provide lower loads compared to the experimental results. It is opposite in regard to the load corresponding with Phase II and Phase III, at least for most of analyzed cases. Relative error of these estimates is generally less than ca. 10% (Table 8). Exception should be made for the case of Phase I load estimation for highly deformable PM polyurethane. One should remember, however, that proposed method of experimental estimation of the Phase I load and deflection is based not on direct crack observation but rather on recorded changes in instantaneous flexural stiffness—this may be partially the source of the observed discrepancies.
- In Phases II and III, the analytical method maintained good agreement with the experimental results for all analyzed adhesives, with load evaluation relative errors remaining below ca 10%.
- The height of the cross-section zone excluded from concrete calculations after cracking corresponded well to the crack height observed in the numerical model for all FPUs (Figure 21, Figure 22 and Figure 23). However, this consistency is lower in the epoxy case (Figure 20), where the numerical solution presented some shear cracks of increased height.
- Theoretical and numerical estimates of maximal normal compressive stresses in concrete and tensile stresses in reinforcement are in good agreement (Table 9 and Figure 18 and Figure 19). Relative difference between these two estimates is less than 10% for all FPUs. In the case of the stiff epoxy adhesive, the analytical results deviated in slightly greater extent from the numerical solution for the cracked beam in Phase III, in which the height of the working concrete zone in the lower beam reached zero (Figure 20), which brings into question the validity of using a method based on Bernoulli’s hypothesis of plane cross-sections.

## 5. Conclusions

This article evaluated the suitability of applying a simple analytical method, including explicit formulas for deflections and maximum stresses, for the assessment of bending of composite reinforced concrete beams joined with either a thin and stiff adhesive layer or a thicker flexible highly deformable bondline. These formulas—derived from a linear model of multi-layer composite beams and based on the assumptions that bent layers work as Bernoulli–Euler beams and sheared adhesive layers work in a simple shear state—formed the basis of an iterative algorithm that accounts for the reduction in the flexural stiffness of a cracked reinforced concrete cross-section. The results obtained in the scenario of the four-point bending of a simply supported beam were compared with experimental results as well as with solutions derived from a non-linear three-dimensional finite element model (FEM) that incorporates non-linear constitutive relationships for adhesives and concrete. Calculations were performed for four different adhesives, analyzing the impact of their stiffness on the accuracy of the analytical results. Based on the research conducted in this study, the following general conclusions can be drawn:

- The results obtained using the analytical method showed good agreement both with the experimental results as well as with the FEM results within the range of service loads. The maximum relative errors were generally below 10%.
- The assumption of a uniform reduction in flexural stiffness for the entire cracked beam resulted in conservative determination of the load-bearing capacity with the analytical model.
- The novelty of the proposed approach is the use of theoretical formulas to quickly estimate deformations and stresses in structures, providing a faster alternative to time-consuming FEM calculations. This method allows for preliminary assessment of a structure’s condition—particularly in the design phase—delivering approximate results that can serve as a basis for more detailed analyses. The proposed approach is especially useful for enabling rapid evaluations of load-bearing capacity in the serviceability limit state—such as in preliminary design or technical inspections—thereby enhancing the efficiency of the design process.

Based on the obtained results, it can be concluded that the proposed analytical method, formulated using closed-form expressions, offers a reliable first approximation of deformation and stress distributions in adhesively bonded reinforced concrete beams. Since the explicit formulas for deflections and stresses can be derived for different support and loading schemes of the composite girders [50], the approach shows potential as a practical design tool, allowing for rapid and straightforward estimation of the required dimensions of composite members. It provides a viable alternative to detailed and computationally demanding FEA procedures, particularly in cases where the overall geometry of the structure is not yet well defined.

The results obtained fit into a broader ongoing research agenda. Investigations into various aspects of the performance of flexible polyurethane joints are currently underway at the Cracow University of Technology in Poland. Significant results have already been achieved with brick-to-brick, FRP-to-brick, and FRP-to-concrete FPU joints. The authors are also involved in detailed studies on the performance of flexible polyurethane joints in timber structures, examining their durability, static, and dynamic responses. Present research involves examining concrete-to-concrete connections in bent elements. Overall, the research reported in this article aims to create a catalog of typical bridge structures featuring polyurethane flexible joints as adhesive layers between precast elements—it is one among multiple branches of future research devoted to examination, description and application of FPU joints.

## Figures and Tables

**Table 1:** Material properties of investigated adhesives.

Parameter	Unit	E	PS	PST	PM
Young’s modulus *E*_a_	MPa	12,800	14	10	4.2
Tensile strength	MPa	28	2.8	3.5	1.3
Ultimate strain	%	0.22	0.30	0.75	1.00

**Table 2:** CDP model parameters.

Dilation Angle [°]	Eccentricity [-]	fb0fc0	K [-]	Viscosity Parameter
37	0.1	1.16	0.667	0.0001

**Table 3:** Compressive and tensile post-critical CDP data.

**Compressive Behavior**		
**Crushing Strain [-]**	**Stress [MPa]**	**Compressive Damage [-]**
0	24.01	0
0.000218	28.23	0
0.000464	34.44	0
0.000805	37.60	0
0.001013	38.00	0
0.001503	36.37	0.043
0.002436	27.65	0.272
0.003194	17.46	0.541
0.004067	3.63	0.905
**Tensile Behavior**		
**Cracking Strain [-]**	**Stress [MPa]**	**Tensile Damage [-]**
0	2.9	0
0.00016	0.824	0.715
0.00028	0.538	0.814
0.00069	0.274	0.905
0.00109	0.195	0.933
0.002	0.130	0.955
0.01	0.041	0.986

**Table 4:** Initial tangent Young’s moduli (in MPa) derived from uniaxial tension tests applied in the analytical model for FPUs.

$\dot{\varepsilon }$	PM	PS	PST
1000%/min	10.33	27.97	16.29
100%/min	7.25	26.72	16.35
10%/min	5.51	25.77	15.96
**1%/min**	**5.36**	**24.53**	**15.04**
0.1%/min	4.73	24.10	14.88

**Table 5:** Analytical solution results for deflections, applied loads, maximal compressive stresses in concrete, and maximal tensile stresses in reinforcement.

Adhesive		Phase I	Phase II	Phase III
Epoxy	w [mm]	0.36	4.00	8.00
	P[kN]	6.32	20.16	40.30
	$\sigma_{c}^{c}\;[MPa]$	2.90	16.6	32.61
	$\sigma_{t}^{s}\;[MPa]$	13.60	227.5	451.6
PS	w [mm]	0.61	4.00	8.00
	P[kN]	4.06	12.2	24.32
	$\sigma_{c}^{c}\;[MPa]$	2.90	12.68	24.31
	$\sigma_{t}^{s}\;[MPa]$	11.05	133.1	266.8
PST	w [mm]	0.63	4.00	8.00
	P[kN]	3.70	10.18	20.26
	$\sigma_{c}^{c}\;[MPa]$	2.90	11.79	22.65
	$\sigma_{t}^{s}\;[MPa]$	10.72	119.01	236.94
PM	w [mm]	0.66	4.00	8.00
	P[kN]	3.24	7.24	14.4
	$\sigma_{c}^{c}\;[MPa]$	2.90	10.27	19.56
	$\sigma_{t}^{s}\;[MPa]$	10.28	105.5	208.19

**Table 6:** FEM solution results for deflection, applied loads, maximal compressive stresses in concrete, and maximal tensile stresses in reinforcement.

Adhesive		Phase I	Phase II	Phase III
Epoxy	w [mm]	0.36	4.00	8.00
	P[kN]	6.46	23.98	40.70
	$\sigma_{c}^{c}\;[MPa]$	3.01	17.4	27.5
	$\sigma_{t}^{s}\;[MPa]$	14.3	207	374
PS	w [mm]	0.53	4.00	8.00
	P[kN]	3.64	14.2	26.0
	$\sigma_{c}^{c}\;[MPa]$	2.70	13.5	23.9
	$\sigma_{t}^{s}\;[MPa]$	11.0	130.0	245
PST	w [mm]	0.57	4.00	8.00
	P[kN]	3.44	10.8	20.8
	$\sigma_{c}^{c}\;[MPa]$	2.79	11.6	20.6
	$\sigma_{t}^{s}\;[MPa]$	11.0	124	227
PM	w [mm]	0.61	4.00	8.00
	P [kN]	3.00	8.32	15.52
	$\sigma_{c}^{c}\;[MPa]$	2.83	10.3	18.7
	$\sigma_{t}^{s}\;[MPa]$	10.9	110.0	202

**Table 7:** Experimental results for deflection and applied loads.

Adhesive		Phase I	Phase II	Phase III
Epoxy	w [mm]	n/a	4.00	8.00
	P [kN]	n/a	22.38	43.82
PS	w [mm]	0.77	4.00	8.00
	P [kN]	3.89	12.74	23.60
PST	w [mm]	0.76	4.00	8.00
	P [kN]	3.60	10.74	18.94
PM	w [mm]	0.77	4.00	8.00
	P [kN]	2.42	7.80	14.12

**Table 8:** Percentage comparison of applied loads obtained from theoretical methods (analytical and numerical) relative to experimental results.

Adhesive		Phase I	Phase II	Phase III
Epoxy	$P_{anal}/P_{exp}$	n/a	90%	92%
	$P_{FEM}/P_{exp}$	n/a	107%	93%
PS	$P_{anal}/P_{exp}$	104%	96%	103%
	$P_{FEM}/P_{exp}$	94%	112%	110%
PST	$P_{anal}/P_{exp}$	102%	95%	107%
	$P_{FEM}/P_{exp}$	96%	110%	115%
PM	$P_{anal}/P_{exp}$	134%	93%	102%
	$P_{FEM}/P_{exp}$	124%	107%	110%

**Table 9:** Percentage comparison of analytical results relative to FEM results for maximal compressive stresses in concrete, and maximal tensile stresses in reinforcement.

Adhesive		Phase I	Phase II	Phase III
Epoxy	$\;\sigma_{c\;anal}^{c}/\sigma_{c\;FEM}^{c}$	96%	95%	119%
	$\sigma_{s\;anal}^{t}/\sigma_{s\;FEM}^{t}$	95%	110%	121%
PS	$\sigma_{c\;anal}^{c}/\sigma_{c\;FEM}^{c}$	107%	94%	102%
	$\sigma_{s\;anal}^{t}/\sigma_{s\;FEM}^{t}$	100%	102%	109%
PST	$\sigma_{c\;anal}^{c}/\sigma_{c\;FEM}^{c}$	103%	102%	110%
	$\sigma_{s\;anal}^{t}/\sigma_{s\;FEM}^{t}$	97%	97%	104%
PM	$\sigma_{c\;anal}^{c}/\sigma_{c\;FEM}^{c}$	104%	102%	110%
	$\sigma_{s\;anal}^{t}/\sigma_{s\;FEM}^{t}$	97%	96%	104%

## Data Availability

The original contributions presented in this study are included in the article. Further inquiries can be directed to the corresponding author.
